# Age at natural menopause and risk of diabetes in adult women: Findings from the China Kadoorie Biobank study in the Zhejiang area

**DOI:** 10.1111/jdi.12775

**Published:** 2017-12-05

**Authors:** Meng Wang, Ru‐Ying Hu, Hao Wang, Wei‐Wei Gong, Chun‐Mei Wang, Kai‐Xu Xie, Zheng‐Ming Chen, Yu Guo, Min Yu, Li‐Ming Li

**Affiliations:** ^1^ Department of NCDs Control and Prevention Zhejiang Provincial Center for Disease Control and Prevention Hangzhou China; ^2^ Tongxiang Center for Disease Control and Prevention Tongxiang China; ^3^ Clinical Trial Service Unit and Epidemiological Studies Unit (CTSU) Nuffield Department of Population Health University of Oxford Oxford UK; ^4^ Chinese Academy of Medical Sciences Beijing China; ^5^ Department of Epidemiology School of Public Health Peking University Health Science Center Beijing China

**Keywords:** Age, Diabetes, Menopause

## Abstract

**Aims/Introduction:**

There has been considerable professional debate on the association between age at menopause and diabetes risk, while the findings are controversial. The present study explored the association between late menopause and the prevalence of diabetes in the Chinese population.

**Material and Methods:**

The data were part of the baseline survey of China Kadoorie Biobank from Zhejiang Province. A total of 17,076 postmenopausal women were included in the present study. Logistic regression models were used to calculate the adjusted odds ratios and their 95% confidence intervals.

**Results:**

Of the participating women, 1,288 (7.54%) had type 2 diabetes. In comparison with those with menopause at 46–52 years, women with menopause at a later age (≥53 years) were 1.21‐fold (95% confidence interval 1.03–1.43) more likely to have diabetes.

**Conclusions:**

The present findings suggested that later age at menopause was associated with an increased prevalence of diabetes.

## Introduction

Diabetes has been growing rapidly worldwide. According to World Health Organization estimates, the global number of people with diabetes has risen from 108 million in 1980 to 422 million in 2014^1^. In China in 2010, the prevalence of diabetes among adults was estimated to be 11.6%, indicating that 113.9 million adults were diagnosed with diabetes^2^. Within the Chinese population, epidemiological studies have shown that the prevalence of diabetes increases with increasing age^2^, ^3^, and in particular, women aged in their 60s and 70s have a higher prevalence than men^3^. Furthermore, a recent nationwide prospective study of 0.5 million Chinese adults showed that diabetes was significantly associated with increased all‐cause mortality risk, which is greater in women than men aged >60 years^4^. Given the higher diabetes‐associated risk in women with older age, it is important to explore some factors unique to women of these age groups.

Menopause is the time in most women's lives when menstrual periods stop permanently. Possibly due to different hormonal environments, the indicator of menopausal age might have an important influence on the health of women. Numerous studies have shown that an early age at menopause is associated with a greater risk of cardiovascular disease^5^, osteoporosis^6^, all‐cause mortality^5^ and depression in later life^7^. In contrast, late age at menopause has shown an increased risk of breast^8^, endometrial^9^ and ovarian cancer^10^.

Whether age at menopause is associated with diabetes risk has also been investigated with conflicting results. Both the European Prospective Investigation into Cancer and Nutrition (EPIC)‐InterAct study in Europe^11^ and Dongfeng‐Tongji cohort study in China^12^ have found that early menopause is associated with a greater risk of type 2 diabetes. Several other studies from the USA and China^13^, ^14^, ^15^ reported that women with late menopause had an increased diabetes risk. Meanwhile, three studies from Italy, Japan and China found no effects of age at menopause on diabetes risk^16^, ^17^, ^18^.

In the current study, we investigated the association of age at natural menopause and diabetes risk in the Zhejiang area. The present cross‐sectional study is part of the China Kadoorie Biobank (CKB) study, which provides us an opportunity to study the association.

## Methods

### Study design and participants

The present cross‐sectional study is part of the survey of the CKB study, which was carried out in Tongxiang, Zhejiang Province, between August 2004 and January 2008. Relevant information about the CKB study is described in detail elsewhere^19^, ^20^, ^21^. In total, 57,704 participants, including 33,677 women (58.36%) aged 30–79 years were recruited in the survey. Of these 33,677 women, 16,601 were excluded for the reason that data on menopause were lacking, they were premenopausal or perimenopausal, or they reported surgical menopause. The relevant data on sociodemographic factors, lifestyle factors, women's reproductive history and medical history were collected face‐to face with a computerized questionnaire. Physical measurements were undertaken by trained health workers. Written informed consent was obtained from all participants.

### Diagnosis of diabetes

In the present study, type 2 diabetes was defined as a random blood glucose level ≥11.1 mmol/L or a fasting blood glucose ≥7.0 mmol/L or a self‐reported history of physician‐diagnosed diabetes.

### Age at natural menopause

Women were asked, ‘Have you had your menopause.’ The options were ‘No,’’ ‘Yes, currently’ and ‘Yes, had menopause.’ If the answer was ‘Yes, had menopause,’ they were then further asked their age at completion of menopause. Women who had hysterectomy or ovariosteresis (unilateral or bilateral) were excluded to avoid surgical menopause.

### Assessment of covariates

Age, marriage status (married, not married), education level (no formal school, primary school, middle school, high school or above), occupation (agriculture and related, factory worker, unemployed or retired, others), household income (<20,000 Yuan, 20,000–35,000 Yuan, ≥35,000 Yuan), parity (≤1, 2, ≥3), smoking category (never, ex‐regular, occasional, current regular), alcohol category (never, ex‐regular, occasional, current regular), family history of diabetes (yes, no) current/ever use of contraceptive (yes, no) and hypertension status (yes, no) were collected face‐to‐face with a computerized questionnaire. Height, weight and waist circumference were obtained by physical examination by trained health workers. Body mass index (BMI; kg/m^2^) was calculated as weight divided by the square of height, and was categorized as obesity (≥28.0), overweight (24.0–27.9), normal weight (18.5–23.9) and underweight (<18.5). Sedentary activity was assessed by reporting of hours watching television or reading per week. To quantify the amount of physical activity, metabolic equivalent tasks were used.

### Statistical analysis

Mean ± standard deviation and percentages were used to describe the continuous and categorical variables. Continuous variables were compared by the Kruskal–Wallis test, and categorical variables were compared using the linear‐by‐linear association χ^2^‐test. The association between age at natural menopause and type 2 diabetes was explored in a series of multivariate logistic regression, and the estimated effect was reported by odds ratios (ORs) with their 95% confidence intervals (CIs). We categorized age at menopause into three groups (≤44, 45–52 and ≥53 years) for the analysis based on the 10th and 90th percentile, and the reference group was aged 45–52 years. In order to adjust for the potential confounders, logistic regression was carried out in four models, which was age in model 1. In model 2, additional factors of marriage status, education level, occupation, household income, parity, family history of diabetes, smoking status, alcohol consumption, sedentary/physical activity and use of contraceptives were included. In model 3, additional factors of the physical measurements of BMI and waist circumference were included. Model 4 adjusted for model 3 plus the diagnosed hypertension. Subgroup analyses were carried out stratified by BMI (obese/overweight, normal/underweight), smoking (yes, no), physical activity (low, middle, high) and use of contraceptives (yes, no). Furthermore, sensitivity analyses were carried out to exclude women with diagnosis of cardiovascular disease or cancer, as well as with both. All analyses were carried out using the SAS statistical package (version 9.2; SAS Institute, Inc., Cary, North Carolina, USA).

## Results

The mean ± standard deviation menopausal age among the 17,076 natural postmenopausal women studied was 48.94 ± 3.83 years. A total of 1,288 (7.54%) women had type 2 diabetes, among which, 712 (55.28%) were diagnosed by self‐reported history and 576 (44.72%) were diagnosed based on the definite glucose values. The baseline characteristics of participating women according to age at menopause were described in Table 1. Women with a later menopausal age were more likely to have an older age (*P* < 0.001), higher parity (*P* < 0.001), higher BMI (*P* < 0.001) and waist circumference (*P* < 0.001) values, longer time of sedentary activity (*P* = 0.015) and diagnosed hypertension (*P* < 0.001). In contrast, women with an earlier menopausal age tended to have lower education levels (*P* < 0.001) and be a current regular smoker (*P* < 0.001). Table 2 showed the results of multivariable models exploring the associations of age at natural menopause and risk of type 2 diabetes. In comparison with those with menopause at the age of 46–52 years, women with natural menopause at a later age (≥53 years) were 1.25‐fold (95% CI: 1.07–1.46) more likely to have diabetes after adjustment for age. With further adjustments for other confounders, the association was unchanged and the adjusted ORs were 1.25 (95% CI: 1.06–1.47) and 1.21 (95% CI: 1.03–1.43) in model 2 and model 3, respectively. However, after adjustment for the diagnosed hypertension, the OR for diabetes was attenuated and tended to be null (1.17, 95% CI: 0.99–1.39). Subgroup analyses showed that the association between later age at natural menopause and diabetes did not differ by BMI, smoking, physical activity and use of contraceptives. In addition, no interaction was observed with any of the variables (Table 3). The sensitivity analyses showed that the ORs for diabetes were largely unchanged after excluding women with cardiovascular disease or cancer, as well as with both (Table 4).

**Table 1:** Baseline characteristics of women according to age at natural menopause

Variable	Age at natural menopause			*P* a
	≤44 years	45–52 years	≥53 years	
Sample size, *n* (%)	1,733 (10.15)	12,991 (76.08)	2,352 (13.77)	
Age (years)	59.47 ± 8.35	58.83 ± 6.77	59.79 ± 5.02	<0.001
Marriage status, *n* (%)				
Married	1,471 (84.88)	11,215 (86.33)	2,038 (86.65)	0.130
Unmarried	262 (15.12)	1,776 (13.67)	314 (13.35)	
Education level, *n* (%)				
No formal school	1,335 (77.03)	9,608 (73.96)	1,621 (68.92)	<0.001
Primary school	324 (18.70)	2,894 (22.78)	602 (25.59)	
Middle school	57 (3.29)	403 (3.10)	102 (4.34)	
High school or above	17 (0.98)	86 (0.66)	27 (1.15)	
Occupation, *n* (%)				
Agriculture and related	1,136 (65.55)	8,202 (63.13)	1,534 (65.22)	0.805
Factory worker	149 (8.60)	1,069 (8.23)	175 (7.44)	
Unemployed/retired	60 (3.46)	696 (5.36)	150 (6.38)	
Others	388 (22.39)	3,024 (23.28)	493 (20.96)	
Household income, *n* (%)				
<20,000 Yuan	503 (29.03)	3,369 (25.93)	610 (25.94)	0.139
20,000–35,000 Yuan	652 (37.62)	5,173 (39.82)	935 (39.75)	
≥35,000 Yuan	578 (33.35)	4,449 (34.25)	807 (34.31)	
Parity, *n* (%)				
≤1	172 (9.92)	961 (7.40)	75 (3.19)	<0.001
2	653 (37.68)	5,718 (44.02)	966 (41.07)	
≥3	879 (50.72)	6,218 (47.86)	1,299 (55.23)	
Smoking category, *n* (%)				
Never	1,652 (95.33)	12,515 (96.34)	2,293 (90.56)	<0.001
Ex‐regular	9 (0.52)	86 (4.96)	11 (0.44)	
Occasional	11 (0.63)	121 (0.93)	15 (0.59)	
Current regular	61 (3.52)	269 (2.07)	33 (1.31)	
Alcohol category, *n* (%)				
Never	1,544 (89.09)	11,549 (88.90)	2,090 (88.86)	0.929
Ex‐regular	9 (0.52)	73 (0.56)	12 (0.51)	
Occasional	123 (7.10)	957 (7.37)	187 (7.95)	
Current regular	57 (3.29)	412 (3.17)	63 (2.68)	
Body mass index (kg/m^2^)	22.35 ± 3.38	22.77 ± 3.38	23.17 ± 3.30	<0.001
Waist circumference (cm)	74.73 ± 9.53	75.75 ± 9.46	76.76 ± 9.41	<0.001
Sedentary activity (h/week)	9.83 ± 7.69	10.48 ± 8.18	10.49 ± 8.07	0.015
Physical activity, *n* (%)				
Low	443 (25.56)	3,585 (27.59)	614 (26.11)	0.383
Middle	829 (47.84)	6,186 (47.62)	1,160 (49.32)	
High	461 (26.60)	3,220 (24.79)	578 (24.57)	
Family history of diabetes, *n* (%)				
No	1,527 (88.11)	11,711 (90.15)	2,150 (91.41)	0.727
Yes	51 (2.94)	424 (3.26)	69 (2.93)	
Current/ever use of contraceptive *n* (%)				
No	1,451 (83.73)	10,513 (80.93)	1,926 (81.89)	0.251
Yes	282 (16.27)	2,478 (19.07)	426 (18.11)	
Hypertension, *n* (%)				
No	1,451 (83.73)	10,411 (80.14)	1,761 (74.87)	<0.001
Yes	282 (16.27)	2,580 (19.86)	591 (25.13)	

a Continuous variables were compared by the Kruskal–Wallis test. Categorical variables were compared using the linear‐by‐linear association χ^2^‐test.

**Table 2:** Adjusted odds ratios (95% confidence intervals) for diabetes according to age at natural menopause

	Total/cases	Model 1	Model 2	Model 3	Model 4
Age at menopause					
≤44 years	1,733/125	0.96 (0.79–1.17)	1.00 (0.81–1.23)	1.03 (0.83–1.27)	1.06 (0.86–1.31)
45–52 years	12,991/948	Ref.	Ref.	Ref.	Ref.
≥53 years	2,352/215	1.25 (1.07–1.46)†	1.25 (1.06–1.47)†	1.21 (1.03–1.43)†	1.17 (0.99–1.39)

Model 1 only adjusted for age; model 2 adjusted for model 1 plus socioeconomic status and health behaviors including marriage status, education level, occupation, household income, parity, family history of diabetes, smoking status, alcohol consumption, sedentary/physical activity and use of contraceptive; model 3 adjusted for model 2 plus the physical measurements of body mass index and waist circumference; model 4 adjusted for model 3 plus hypertension. ^†^Significant results.

**Table 3:** Adjusted odds ratios (95% confidence intervals) for diabetes stratified by body mass index, smoking, physical activity and use of contraceptives

	Total/cases	Adjusted OR (95% CI)†	*P*‐value for interaction‡
Overall	17,076/1,288	1.21 (1.03–1.43)	
BMI			
Obese/overweight	5,918/581	1.05 (0.82–1.35)	0.30
Normal/underweight	11,158/707	1.35 (1.08–1.68)	
Smoking			
Yes	616/57	1.32 (0.49–3.59)	0.81
No	16,460/1231	1.20 (1.02–1.42)	
Physical activity			
Low	4,642/507	1.40 (1.07–1.83)	0.52
Middle	8,175/563	1.15 (0.90–1.47)	
High	4,259/218	1.09 (0.72–1.65)	
Use of contraceptives			
Yes	3,186/209	1.15 (0.76–1.73)	1.00
No	13,890/1,079	1.21 (1.01–1.46)	

^†^Model 3: adjusted for age, marriage status, education level, occupation, household income, parity, family history of diabetes, smoking status, alcohol consumption, sedentary/physical activity, use of contraceptive, physical measurements of body mass index (BMI) and waist circumference. ^‡^Effect modification was tested by adding interaction terms between these variables (BMI, smoking, physical activity, use of contraceptive) and age at natural menopause to the model. CI, confidence interval; OR, odds ratio.

**Table 4:** Sensitivity analyses: Adjusted odds ratios (95% confidence intervals) for diabetes according to age at natural menopause

Age at menopause	Total/cases	Model 1	Model 2	Model 3	Model 4
Excluding women with cardiovascular disease (stroke/transient ischemic attacks, coronary heart disease)					
≤44 years	1,708/121	0.96 (0.79–1.17)	0.98 (0.80–1.21)	1.01 (0.82–1.25)	1.04 (0.84–1.28)
45–52 years	12,744/917	Ref.	Ref.	Ref.	Ref.
≥53 years	2,303/206	1.24 (1.06–1.45)†	1.24 (1.05–1.47)†	1.21 (1.02–1.43)†	1.17 (0.99–1.39)
Excluding women with cancer					
≤44 years	1,728/124	0.96 (0.79–1.17)	1.00 (0.81–1.23)	1.02 (0.83–1.26)	1.05 (0.85–1.30)
45–52 years	12,952/942	Ref.	Ref.	Ref.	Ref.
≥53 years	2,346/214	1.26 (1.07–1.47)†	1.25 (1.06–1.48)†	1.22 (1.03–1.44)†	1.18 (0.99–1.39)
Excluding women with cardiovascular disease and cancer					
≤44 years	1,703/120	0.96 (0.79–1.17)	0.98 (0.79–1.21)	1.01 (0.81–1.24)	1.03 (0.83–1.28)
45–52 years	12,705/911	Ref.	Ref.	Ref.	Ref.
≥53 years	2,297/205	1.24 (1.06–1.46)†	1.25 (1.05–1.47)†	1.21 (1.02–1.43)†	1.17 (0.99–1.39)

Model 1 only adjusted for age; model 2 adjusted for model 1 plus socioeconomic status and health behaviors including marriage status, education level, occupation, household income, parity, family history of diabetes, smoking status, alcohol consumption, sedentary/physical activity and use of contraceptives; model 3 adjusted for model 2 plus the physical measurements of body mass index and waist circumference; model 4 adjusted for model 3 plus hypertension. ^†^Significant results.

## Discussion

In the present cross‐sectional study, we found that later age at menopause influenced the prevalence of type 2 diabetes in Chinese women. The risk of type 2 diabetes was 21% higher in women with menopause after the age of 53 years compared with women having their menopause at the age of 45–52 years. The association remained significant after adjustment for a wide range of potential confounders, and the effect estimates were robust to exclude women with cardiovascular disease or cancer, as well as women with both. In addition, subgroup analyses showed that the association was not modified by BMI, smoking, physical activity and use of contraceptives. The present study is probably one of the few studies showing that late menopause might increase the risk of diabetes among Asian postmenopausal women.

Consistent with previous studies showing that late menopause can increase the diabetes risk^13^, ^14^, ^15^, the present findings also showed that the age at natural menopause affected type 2 diabetes risk even after adjustment for age, BMI and other potential confounders, though the cut‐off points for menopausal age differed from the previous studies. Another two studies involving menopausal age ≥53 years, which was in line with our cut‐off point for later age at menopause in the present study, showed that the association was not statistically significant after multivariable adjustment^12^, ^16^. However, it is worth noting that some other studies examining the association between age at menopause and diabetes have yielded completely contradictory results, showing that early menopause is associated with a greater risk of type 2 diabetes^11^, ^12^. Most recently, Muka *et al*.^22^, in a prospective, population‐based study, confirmed the increased risk of type 2 diabetes with early onset menopause in postmenopausal women. The reasons for the inconsistent results were unknown and possibly as a result of differences in participant characteristics, study design, sample size and menopausal age grouping. Additional prospective studies investigating the effects of age at menopause on diabetes risk are warranted. In addition, the significant association of later age at menopause and diabetes in the present study tended to be null after adjustment for hypertension. Similarly, in a previous study, after adjusting for hypertension and blood lipid, the effect of postmenopausal status on the risk of type 2 diabetes was attenuated^23^. The findings along with the present results suggested that when investigating the menopause‐diabetes association in further studies, the metabolic factors should be also considered.

Although mechanisms accounting for the association between late menopause and increased diabetes risk among postmenopausal women are unclear, recent studies raised the possibility that the changes in hormones, as well as body composition, play an important role. First, later age at menopause might lead to prolonged endogenous estrogen exposure, and increasing evidence has shown that high endogenous estrogen levels were linked to an increased risk of impaired fasting glucose and diabetes in postmenopausal women^24^, ^25^. Second, with 128,000 postmenopausal women, a recent CKB study provided reliable evidence that later age at menopause was independently associated with increased adiposity^26^. According to available evidence, changes in body fat distribution around the time of menopause could influence the diabetes risk with decreasing tissue insulin sensitivity and glucose tolerance^27^, ^28^, ^29^, ^30^. Further research is required to unravel the related mechanisms by which late menopause might affect diabetes risk in postmenopausal women.

The strengths of the present study included the large sample size and robust results after adjustment for various covariates. However, several limitations should be considered in this study. First, with self‐reported onset age at menopause, misclassification might have occurred. However, menopausal age by recall showed high correlations between two interviews^31^, ^32^, ^33^. Second, despite adjustment for a comprehensive set of potential confounders, we cannot rule out residual confounding from other known risk factors for diabetes, such as sleep duration and age at menarche. Third, in the self‐reported questionnaire, we did not ask about the cause for menopause, and to avoid surgical menopause, we just excluded women with hysterectomy or ovariosteresis (unilateral or bilateral). This might lead to misclassification and slightly exaggerate the sample of women with natural menopause in the present study.

In conclusion, in the present study of a large number of Chinese postmenopausal women, later age at menopause was associated with an increased prevalence of diabetes.

## Disclosure

The authors declare no conflict of interest.

## References

[1] WHO . Diabetes. Available from: http://www.who.int/mediacentre/factsheets/fs312/en/ Accessed June 26, 2017.

[2] Xu Y , Wang L , He J , *et al* Prevalence and control of diabetes in Chinese adults. JAMA 2013; 310: 948–959. 24002281 10.1001/jama.2013.168118

[3] Yang W , Lu J , Weng J , *et al* Prevalence of diabetes among men and women in China. N Engl J Med 2010; 362: 1090–1101. 20335585 10.1056/NEJMoa0908292

[4] Bragg F , Holmes MV , Iona A , *et al* Association between diabetes and cause‐specific mortality in rural and urban areas of China. JAMA 2017; 317: 280–289. 28114552 10.1001/jama.2016.19720 PMC6520233

[5] Muka T , Oliver‐Williams C , Kunutsor S , *et al* Association of age at onset of menopause and time since onset of menopause with cardiovascular outcomes, intermediate vascular traits, and all‐cause mortality: a systematic review and meta‐analysis. JAMA Cardiol 2016; 1: 767–776. 27627190 10.1001/jamacardio.2016.2415

[6] Cauley JA , Danielson ME , Greendale GA , *et al* Bone resorption and fracture across the menopausal transition: the Study of Women's Health Across the Nation. Menopause 2012; 19: 1200–1207. 22850443 10.1097/gme.0b013e31825ae17e PMC3483443

[7] Georgakis MK , Thomopoulos TP , Diamantaras AA , *et al* Association of age at menopause and duration of reproductive period with depression after menopause: a systematic review and meta‐analysis. JAMA Psychiatry 2016; 73: 139–149. 26747373 10.1001/jamapsychiatry.2015.2653

[8] Li H , Sun X , Miller E , *et al* BMI, reproductive factors, and breast cancer molecular subtypes: a case‐control study and meta‐analysis. J Epidemiol 2017; 27: 143–151. 28142040 10.1016/j.je.2016.05.002 PMC5376312

[9] Ali AT . Reproductive factors and the risk of endometrial cancer. Int J Gynecol Cancer 2014; 24: 384–393. 24463639 10.1097/IGC.0000000000000075

[10] Tsilidis KK , Allen NE , Key TJ , *et al* Oral contraceptive use and reproductive factors and risk of ovarian cancer in the European Prospective Investigation into Cancer and Nutrition. Br J Cancer 2011; 105: 1436–1442. 21915124 10.1038/bjc.2011.371 PMC3241548

[11] Brand JS , van der Schouw YT , Onland‐Moret NC , *et al* Age at menopause, reproductive life span, and type 2 diabetes risk: results from the EPIC‐InterAct study. Diabetes Care 2013; 36: 1012–1019. 23230098 10.2337/dc12-1020 PMC3609516

[12] Shen L , Song L , Li H , *et al* Association between earlier age at natural menopause and risk of diabetes in middle‐aged and older Chinese women: the Dongfeng‐Tongji cohort study. Diabetes Metab 2017; 43: 345–350. 28129998 10.1016/j.diabet.2016.12.011

[13] LeBlanc ES , Kapphahn K , Hedlin H , *et al* Reproductive history and risk of type 2 diabetes mellitus in postmenopausal women: findings from the Women's Health Initiative. Menopause 2017; 24: 64–72. 27465714 10.1097/GME.0000000000000714 PMC5477993

[14] Yang A , Liu S , Cheng N , *et al* Reproductive factors and risk of type 2 diabetes in an occupational cohort of Chinese women. J Diabetes Complications 2016; 30: 1217–1222. 27345735 10.1016/j.jdiacomp.2016.06.011

[15] Fu Y , Yu Y , Wang S , *et al* Menopausal Age and chronic diseases in elderly women: a cross‐sectional study in Northeast China. Int J Environ Res Public Health 2016; 13: 936. 10.3390/ijerph13100936 PMC5086675 27669270

[16] Di Donato P , Giulini NA , Bacchi Modena A , *et al* Risk factors for type 2 diabetes in women attending menopause clinics in Italy: a cross‐sectional study. Climacteric 2005; 8: 287–293. 16397927 10.1080/13697130500196866

[17] Lee JS , Hayashi K , Mishra G , *et al* Independent association between age at natural menopause and hypercholesterolemia, hypertension, and diabetes mellitus: Japan nurses’ health study. J Atheroscler Thromb 2013; 20: 161–169. 23079582 10.5551/jat.14746

[18] Qiu C , Chen H , Wen J , *et al* Associations between age at menarche and menopause with cardiovascular disease, diabetes, and osteoporosis in Chinese women. J Clin Endocrinol Metab 2013; 98: 1612–1621. 23471979 10.1210/jc.2012-2919

[19] Chen Z , Chen J , Collins R , *et al* China Kadoorie Biobank of 0.5 million people: survey methods, baseline characteristics and long‐term follow‐up. Int J Epidemiol 2011; 40: 1652–1666. 22158673 10.1093/ije/dyr120 PMC3235021

[20] Chen Z , Lee L , Chen J , *et al* Cohort profile: the Kadoorie Study of Chronic Disease in China (KSCDC). Int J Epidemiol 2005; 34: 1243–1249. 16131516 10.1093/ije/dyi174

[21] Li LM , Lv J , Guo Y , *et al* The China Kadoorie Biobank: related methodology and baseline characteristics of the participants. Zhonghua Liu Xing Bing Xue Za Zhi 2012; 33: 249–255. 22613372

[22] Muka T , Asllanaj E , Avazverdi N , *et al* Age at natural menopause and risk of type 2 diabetes: a prospective cohort study. Diabetologia 2017; 60: 1951–1960. 28721436 10.1007/s00125-017-4346-8 PMC6448832

[23] Heianza Y , Arase Y , Kodama S , *et al* Effect of postmenopausal status and age at menopause on type 2 diabetes and prediabetes in Japanese individuals: Toranomon Hospital Health Management Center Study 17 (TOPICS 17). Diabetes Care 2013; 36: 4007–4014. 24170752 10.2337/dc13-1048 PMC3836104

[24] Golden SH , Dobs AS , Vaidya D , *et al* Endogenous sex hormones and glucose tolerance status in postmenopausal women. J Clin Endocrinol Metab 2007; 92: 1289–1295. 17244779 10.1210/jc.2006-1895

[25] Kalyani RR , Franco M , Dobs AS , *et al* The association of endogenous sex hormones, adiposity, and insulin resistance with incident diabetes in postmenopausal women. J Clin Endocrinol Metab 2009; 94: 4127–4135. 19789205 10.1210/jc.2009-0910 PMC2775652

[26] Yang L , Li L , Millwood IY , *et al* Adiposity in relation to age at menarche and other reproductive factors among 300 000 Chinese women: findings from China Kadoorie Biobank study. Int J Epidemiol 2017; 46: 502–512. 27524817 10.1093/ije/dyw165 PMC5837303

[27] Vryonidou A , Paschou SA , Muscogiuri G , *et al* MECHANISMS IN ENDOCRINOLOGY: Metabolic syndrome through the female life cycle. Eur J Endocrinol 2015; 173: R153–R163. 26034072 10.1530/EJE-15-0275

[28] Sites CK , Calles‐Escandón J , Brochu M , *et al* Relation of regional fat distribution to insulin sensitivity in postmenopausal women. Fertil Steril 2000; 73: 61–65. 10632413 10.1016/s0015-0282(99)00453-7

[29] Barrett‐Connor E , Schrott HG , Greendale G , *et al* Factors associated with glucose and insulin levels in healthy postmenopausal women. Diabetes Care 1996; 19: 333–340. 8729156 10.2337/diacare.19.4.333

[30] Campbell AJ , Busby WJ , Horwath CC , *et al* Relation of age, exercise, anthropometric measurements, and diet with glucose and insulin levels in a population aged 70 years and over. Am J Epidemiol 1993; 138: 688–696. 8237984 10.1093/oxfordjournals.aje.a116906

[31] Cairns BJ , Liu B , Clennell S , *et al* Lifetime body size and reproductive factors: comparisons of data recorded prospectively with self reports in middle age. BMC Med Res Methodol 2011; 11: 7. 21241500 10.1186/1471-2288-11-7 PMC3034712

[32] Rödström K , Bengtsson C , Lissner L , *et al* Reproducibility of self‐reported menopause age at the 24‐year follow‐up of a population study of women in Göteborg. Sweden. Menopause 2005; 12: 275–280. 15879916 10.1097/01.gme.0000135247.11972.b3

[33] Bosetti C , Tavani A , Negri E , *et al* Reliability of data on medical conditions, menstrual and reproductive history provided by hospital controls. J Clin Epidemiol 2001; 54: 902–906. 11520649 10.1016/s0895-4356(01)00362-6

